# Andrographolide nanophytosomes exhibit enhanced cellular delivery and pro-apoptotic activities in HepG2 liver cancer cells

**DOI:** 10.1080/10717544.2023.2174209

**Published:** 2023-02-10

**Authors:** Thikryat Neamatallah, Azizah M. Malebari, Abdulmohsin J. Alamoudi, Syed Nazreen, Mohammad Mahboob Alam, Hawazen H. Bin-Melaih, Osama A. Abuzinadah, Shaimaa M. Badr-Eldin, Gharam Alhassani, Lamar Makki, Mohammed Z. Nasrullah

**Affiliations:** a Department of Pharmacology and Toxicology, Faculty of Pharmacy, King Abdulaziz University, Jeddah, Saudi Arabia; b Department of Pharmaceutical Chemistry, Faculty of Pharmacy, King Abdulaziz University, Jeddah, Saudi Arabia; c Department of Chemistry, Faculty of Science, Albaha University, Albaha, Saudi Arabia; d Department of Biological Sciences, Faculty of Sciences, King Abdulaziz University, Jeddah, Saudi Arabia; e Department of Pharmaceutics, Faculty of Pharmacy, King Abdulaziz University, Jeddah, Saudi Arabia; f Department of Pharmaceutics and Industrial Pharmacy, Faculty of Pharmacy, Cairo University, Cairo, Egypt

**Keywords:** Andrographolide, phytosomes, HepG2 cells, apoptosis, liver cancer

## Abstract

Andrographolide (AG), a major active constituent of *Andrographis paniculata,* is known to hinder proliferation of several types of cancer cells. However, its poor solubility and cellular permeability restrict its use in clinical applications. In this study, AG-loaded phytosomes (AG-PTMs) were formulated and optimized with respect to particle size using l-α-phosphatidylcholine (PC):AG ratio and sonication time (ST) as independent variables. The optimized formula was prepared at 1:2.7 for AG:PC molar ratio and 4.9 min for ST and exhibited a particle size of 243.7 ± 7.3 nm, polydispersity index (PDI) of 0.310 and entrapment efficiency of 72.20 ± 4.53. Also, the prepared formula showed a slow release of AG over 24-h period. The antiproliferative activity of AG-PTMs was investigated against the liver cancer cell line HepG2. AG-PTMs significantly repressed the growth of HepG2 cells with an IC_50_ value of 4.02 ± 0.14 µM. AG uptake by HepG2 cells was significantly enhanced in incubations containing the optimized formula. AG-PTMs also caused G2-M cell cycle phase arrest and increased the fraction of apoptotic cells in pre-G1 phase. These effects were associated with induction of oxidative stress and mitochondrial dysfunction. In addition, AG-PTMs significantly upregulated mRNA expression of *BAX* and downregulated that of *BCL2.* Furthermore, AG-PTMs significantly enhanced the concentration of caspase-3 in comparison to raw AG. These data indicate that the phytosomal delivery of AG significantly inhibited HepG2 cell proliferation through enhanced cellular uptake, arresting cell cycle at the G2-M phase and inducing mitochondrial-dependent apoptosis.

## 1. Introduction

Liver cancer is one of the leading causes of cancer-related mortalities. About 75%–85% of primary liver cancers begin and progress in hepatocytes, which are collectively referred to as hepatocellular carcinoma (K. Wang & Sun, 2018; Sung et al., 2021). Patients with hepatocellular carcinoma are usually asymptomatic in the early stages as symptoms appear later when the disease advances (Llovet et al., 2008). Systemic therapies including antiangiogenic antibodies, immune checkpoint inhibitors and tyrosine kinase inhibitors are recommended only at the advanced stages (Cucarull et al., 2022). Moreover, patients may develop resistance to these treatments or suffer from severe adverse effects (Iavarone et al., 2011; J. Chen et al., 2015; Zhu et al., 2017; Abou-Alfa et al., 2018; Kudo et al., 2018; Lei et al., 2019; Zan et al., 2019). Therefore, these limitations support the discovery and development of new anticancer therapies.

Phytochemicals have been extensively investigated for their anticancer activities exhibiting different mechanisms, making them promising candidates for cancer treatment (Choudhari et al., 2020). Of these compounds, andrographolide (AG) isolated from *Andrographis paniculata,* is a well-known bicyclic diterpenoid lactone that is effective against several cancer cell types such as renal, lung, melanoma, ovarian, colon, liver and prostate cancer cells (Rajagopal et al., 2003; Kumar et al., 2004; Satyanarayana et al., 2004; J. Li et al., 2007; H-P. Chao et al., 2010; Joseph & Joseph, 2016). AG and its potent derivative AGS-30 target cancer cells through certain pathways that include inducing cell cycle arrest, promoting cell apoptosis as well as inhibiting metalloproteinases and growth factors (Malik et al., 2021; Z. Liu et al., 2022). Based on the Biopharmaceutics Classification System (BCS), AG is classified as class II, showing poor solubility (Lee et al., 2019; Yen et al., 2020). Therefore, there has been significant interest in developing an effective delivery system for AG to resolve this significant limitation.

Nanomedicine has sparked a significant interest in terms of enhancing the delivery of several drugs as well as overcoming their pharmacological limitations. The incorporation of phytochemicals into nanocarriers showed improvements in their stability, solubility, bioavailability and tumor-targeting effect (Yan et al., 2016; Qi et al., 2017). Phytosome (PTM) has been used to enhance the anticancer activity of phytochemical compounds (Alhakamy, Badr-Eldin et al., 2020; Alhakamy, Fahmy et al., 2020; Alhakamy et al., 2021; Al-Rabia et al., 2022). PTMs are lipid-based vesicular delivery systems composed of phytochemical compounds and phospholipids (Lu et al., 2019; Alharbi et al., 2021). Different solvents serve as a reaction medium for hydrogen bond formation between the polar functionalities of phytochemicals and the phosphate groups at the polar heads of phospholipids, leading to complete integration of phytochemicals into the vesicular membrane. In this regard, the polar portion of these complexes is encapsulated by two long fatty acid chains, forming a lipophilic surface (Khan et al., 2013; Ghanbarzadeh et al., 2016; Shakeri & Sahebkar, 2016). These formed hydrogen bonds improve the stability of the incorporated phytochemicals, making them more resistant to hydrolysis, oxidation and photolysis (PM. Kidd, 2009; Awasthi et al., 2011). Furthermore, PTMs can enhance the bioavailability of phytochemicals due to the amphipathic nature of their phospholipid components. They allow the passage of phytochemicals from the water phase outside the small intestinal enterocyte to the lipid phase of its outer cell membrane. Thus, enhancing cellular internalization and bioavailability (Bhattacharya, 2009; P. Kidd & Head, 2005).

The present study was designed to formulate AG into PTMs using face-centered central composite design to enhance the antiproliferative and pro-apoptotic activities of AG against the hepatocellular carcinoma cells HepG2.

## 2. Materials and methods

### 2.1. Chemicals

Methanol, chloroform, dimethylsulfoxide (DMSO) and thiazolyl blue tetrazolium bromide (MTT) were purchased from Sigma-Aldrich (Saint Louis, MO, USA). Dulbecco’s modified eagle’s medium (DMEM), phosphate-buffered saline (PBS), TrypLE™ Express Enzyme (1×), penicillin/streptomycin and fetal bovine serum (FBS) were obtained from Gibco (Grand Island, NY, USA). Soybean l-α-phosphatidylcholine (95%) (PC) was purchased from Avanti Polar Lipids, Inc. (Alabaster, AL, USA). Sorafenib (SORA) was purchased from MCE (Med Chem Express, Monmouth Junction, NJ, USA).

### 2.2. Isolation of AG

*Andrographis paniculata* leaves were procured from Delhi, India, and authenticated by Dr. H.B. Singh, taxonomist, NISCAIR, CSIR, New Delhi. Leaves were shade dried and powdered. The extraction of AG was carried out according to the reported method with minor modifications at Al-Baha University, KSA (W-W. Chao & Lin, 2010). The plant powder was cold macerated with dichloromethane:methanol (1:1) overnight and then evaporated under vacuum resulting in a green residue. This residue was treated with a mixture of toluene and petroleum ether to remove chlorophyll. The left residue was then column chromatographed on silica gel using chloroform as a solvent. The AG was obtained at 8% methanol in chloroform. It was crystallized using methanol to afford pure AG (6.5 g). The confirmation was performed by comparing it with the standard andrographolide (98% purity, Sigma Aldrich) and its melting point (lit m.p. 228–229 °C), ^1^H NMR, ^13 ^C NMR and mass spectrometry available in literature (Du et al., 2003; Akowuah et al., 2006; Syukri et al., 2018).

### 2.3. Experimental design and optimization of AG-PTMs

To achieve the aim of enhancing the antiproliferative and pro-apoptotic activities of AG against the hepatocellular carcinoma cells HepG2, the principles of nanotechnology has been applied to yield nanovesicular lipid-based delivery system for AG. For lipid-based delivery systems, it is well reported that particles smaller than 400 nm exhibit preferential distribution within cancerous tissues (Sharma et al., 2014; Yingchoncharoen et al., 2016; Badr-Eldin et al., 2021). However, it is worthy note that inadequate tumor penetration caused by the pathogenic features the tumor evolved could balance the preferred accumulation of nanodelivery systems and their resulting therapeutic efficacy (Zhang et al., 2019). Accordingly, to ensure efficient tumor penetration, the particle size of the developed PTMs was tuned to reduced value via numerical optimization and experimental design. Based on previous literature, the active to phospholipid ratio and sonication time were two formulation factors that could mostly influence the size of phytosomes, while their effects and the interaction between them could vary according to the properties of active used. Increasing lipid content above a certain limit could result in increasing the size, while sonication could decrease the size of vesicular systems in general by the virtue of cavitation forces (Freag et al., 2013; Song et al., 2021; Alhakamy & Fahmy, 2022; X. Chen et al., 2022). Thus, these two variables were selected for studying their effect on the phytosomal size to outline the optimized levels that could achieve the desired reduced size upon combination. Response surface methodology, specifically face-centered central composite design (*α* = 1), was utilized to optimize the proposed AG-PTMs. Andrographolide:phosphatidylcholine molar ratio (*X*_1_, AG:PC) and sonication time (*X*_2_, ST, min) were studied as quantitative independent variables. The upper and lower coded and actual levels of the two variables are compiled in ***Table 1***. Particle size (PS, nm, *Y*) was chosen as the response parameter. As per the selected design, 13 experimental runs, including factorial, axial and five center points were produced by Design-Expert software (Version 12; Stat-Ease Inc., Minneapolis, MN, USA); for every experimental run, the combinations of variables’ levels are listed in ***Table 2***. The optimal model fitting of the measured size was chosen amongst linear, two-factor interaction (2FI), and quadratic models on the basis of the computed predicted and adjusted determination coefficients (*R*^2^) in addition to the predicted residual sum of squares (PRESS). Generated by the software, the diagnostic plots were used to assess the goodness of data fitting. To estimate the significance of the studied variables at *p* < .05 and the interaction between them, analysis of variance (ANOVA) was employed for statistical analysis of the measured size. The terms’ coefficients in the equation conveying the best fitting model were deployed to predict the relative magnitude of the impact of the corresponding variable as well as the interaction between the two variables. Furthermore, the effect of the investigated variables and the interaction between them was graphically illustrated using perturbation, contour and response surface plots.

**Table 1.** Independent variables’ levels (coded and actual) in the central composite design and desirability constraints of the measured size of AG-PTMs.

Factoars	Levels		
	–1	0	+1
*X*1: AG:PC molar ratio	1:1	1:3	1:5
*X*2: Sonication time (min)	1	3	5
Response	Desirability constraints		
*Y*: Particle size (nm)	Minimize		

AG, andrographolide; PTM, Phytosomes; PC, phosphatidyl choline.

**Table 2.** Combination of independent variables’ levels in AG-PTMs experimental runs and their corresponding responses.

Run no.	Independent variables		Response
	AG:PC molar ratio (*X*1)	Sonication time (*X*2, min)	Mean PS (*Y*, nm)
1	1:3	3	319.8 ± 13.2
2	1:5	3	397.9 ± 19.89
3	1:1	5	307.1 ± 13.81
4	1:1	1	569.2 ± 28.30
5	1:3	3	278.1 ± 10.7
6	1:5	5	329.7 ± 13.18
7	1:3	3	309.5 ± 15.45
8	1:3	3	311.8 ± 6.9
9	1:3	5	246.7 ± 7.9
10	1:3	3	299.3 ± 15.9
11	1:1	3	346.9 ± 10.40
12	1:5	1	489.9 ± 19.59
13	1:3	1	448.9 ± 23.79

AG, andrographolide; PTM, Phytosomes; PS, particle size; SD, standard deviation. Results are presented as mean ± SD, n = 5.

Numerical optimization and desirability approach was applied to the measured response to anticipate the optimal variables’ levels that could achieve the desired goal upon the combination, Table 1.

### 2.4. Preparation of AG-PTMs

AG-PTMs were prepared by using by the solvent evaporation method. AG and PC were placed in a 100-mL round-bottom flask in the specified molar ratio according to Design-Expert software and dissolved in 20 mL of chloroform/methanol (1:1 vol/vol). The solvents were evaporated by rotary evaporation (BÜCHI Labortechnik AG, Flawil, Switzerland) until the thin film was produced in the round-bottom flask. Then, placed in a vacuum oven at 40 °C for 24 h to evaporate remaining solvent traces. After that, 20 mL of distilled water was added to reconstitute the formed film. The hydrated AG-PTMs were sonicated by a probe sonicator (Vibra cell, Sonics, Newtown, CT, USA). For separation of un-entrapped active, the resulting dispersion was ultracentrifugated at 20,000 rpm for 10 min at 4 °C (OptimaTM MAX-XP, Beckman Coulter Inc., USA). The residue was washed twice with distilled water and ultracentrifuged again for 10 min. The supernatant was discarded and the residue was lyophilized for 72 h using 2% (wt/vol) dextran solution as a cryoprotectant to obtain AG-PTMs. Blank-PTMs were also prepared under the same condition without adding AG. The prepared formulations were stored at 4 °C for further investigations (Hou et al., 2013).

### 2.5. Particle size measurement

AG-PTMs were diluted with deionized water, then dynamic light scattering technique (DLS) was used to utilized to assess the particle size using a Malvern Zetasizer Nano ZS particle size analyzer (Malvern Instrument, Worcestershire, UK). The data were expressed as the average of six determinations.

### 2.6. Entrapment efficiency

Percentage of AG content in the AG-PTMs was calculated using the following equation:

$$\text{AG entrapment efficiency }(\%)=\frac{\text{AG in the filtrate }}{\text{AG originally added }}X\;100$$

AG was analyzed spectrophotometrically after dilution with methanol–chloroform (1:1) according to the previously described method (Levita, 2014).

### 2.7. Fourier-transform infrared characterization of the optimized AG-PTMs

The optimized AG-PTMs and their formula components including AG, PC and AG plus PC simple mixture were measured in the range of 4000–400 cm^−1^ using a Fourier-transform infrared (FTIR) spectrometer (Tensor 37, Bruker, Fremont, CA, USA).

### 2.8. Ag release assay

The dialysis bag method was used to assess the release pattern of AG from the optimized AG-PTMs formula, where the sample containing 20 mg of AG was loaded into the activated dialysis bag (12 kDa cut-off) and closed both ends hermetically. Thereafter the release of AG from the setup was determined in paddle-type dissolution apparatus, where phosphate buffer saline (pH 7.4) was used as releasing media. The paddle was set at 50 rpm and the temperature of the system was maintained at 37 ± 0.5 °C. Samples were withdrawn at time points 0, 0.5,1,2,4, 6, 8, 12 and 24 h. AG was determined spectrophotometrically as previously described (Levita, 2014).

### 2.9. Pharmacological effects of optimized AG-PTMs on human hepatocellular carcinoma cell line HepG2

#### 2.9.1. Cell culture

Human hepatocellular carcinoma cell line (HepG2) was purchased from American Type Culture Collection (ATCC). Cells were maintained in Eagle’s minimum essential medium (EMEM) supplemented with 100 μg/mL of streptomycin, 100 units/mL of penicillin, and 10% fetal bovine serum (FBS) at 5% CO_2_ and 37 °C.

#### 2.9.2. Determination of IC_50_ using MTT assay

To assess cell viability, MTT assay was carried out after treating HepG2 cells with blank-PTMs, AG and AG-PTMs. SORA was used as a positive control. In 96-well plates, cells were seeded at a density of 2 × 10^3^ cells per well. Blank-PTMs, AG, AG-PTMs or SORA were added to the cells after overnight incubation at different concentrations ranging from 1 to 125 µM for 48 h. Later, the culture medium was then aspirated and replaced with MTT solution (0.5 mg/mL). This was followed by an incubation period of 3 h at 37 °C. Next, the MTT solution was aspirated and replaced with 100 μL DMSO for 5 min to dissolve the formazan crystals. The absorbance was measured at a 570 nm wavelength with a microplate reader (Synergy HT, BioTek, Winooski, VT, USA). The IC_50_ values were calculated using GraphPad Prism software (GraphPad, Inc., La Jolla, CA, USA).

#### 2.9.3. Cellular uptake analysis

HepG2 cells (1 × 10^5^ cells/dish) under presence of 5% CO_2_, at 37 °C were incubated for 2 and 4 h after treatment with AG IC50 values or equivalent concentrations of AG-PTMs. Lysis solution was added for 30 min at 37 °C after monolayers were washed with PBS. The HPLC technique used for analyzing the cell lysates aliquots embraced the use of methanol–water (52:48, v/v) as the mobile phase with a rate of 0.8 mL/min. AG was detected spectrophotometrically at wavelength of 225 nm (Suo et al., 2007).

#### 2.9.4. Cell cycle analysis

Cell cycle phases of HepG2 cells were analyzed using Propidium Iodide (PI) Flow Cytometry Kit (ab139418, Abcam, Cambridge, UK). In six-well plates, cells were seeded at a density of 1 × 10^5^ cells per well and incubated for 24 h. The cells were then treated to the predetermined IC_50_ values for 48 h. After that, the cells were detached and collected via centrifugation at 800 × *g* for 5 min, before being washed and resuspended with 1 mL of PBS. After a second centrifugation at 800 × *g* for 5 min, the cells were fixed with ethanol (70%) and stored at 4 °C for 2 h. After washing with PBS, the pellet was treated with RNase and further stained with 200 µL of PI at 37 °C in dark for 30 min. The cellular DNA content was determined at 488 nm using a FACSCalibur flow cytometer (BD Biosciences, San Jose, CA). Acquired data were examined utilizing the CellQuest Software (BD Bioscience, San Jose, CA, USA).

#### 2.9.5. Annexin V/PI apoptotic assay

The effects of AG, AG-PTMs and SORA on the apoptotic profile of HepG2 cells were determined utilizing Annexin V-FITC Apoptosis Detection Kit (K101, BioVision Research Products, Mountain View, CA, USA). In brief, cells were seeded in six-well plates at a density of 1 × 10^5^ cells per well for 24 h. They were then exposed for 48 h to the predetermined IC_50_ values of each treatment. After that, the cells were harvested by trypsinization, washed with PBS and resuspended in 500 μL of 1X Binding Buffer. Next, they were mixed with 5 μL of Annexin V-FITC on ice and then 5 μL of PI at room temperature for 30 min. Samples were analyzed using the BD accuri flow cytometer (FACSCalibur, BDBiosciences, San Diego, CA, USA) and GraphPad Prism software for analyzing the data.

#### 2.9.6. Caspase-3 assay

Assessment of human active caspase-3 was carried out using Caspase-3 (active) ELISA kit (KHO1091, Thermo Fisher Scientific, Carlsbad, CA, USA). Briefly, HepG2 cells were seeded and treated with a predetermined IC_50_ of AG, AG-PTMs and SORA for 48 h. Then, cells were collected, washed with PBS and lysed with extraction buffer for 30 min on ice with agitation. Lysates were centrifuged for 10 min at 13,000 rpm and clear lysates were transferred into clean microcentrifuge tubes. For the assay, an aliquot of 100 µL of standards and samples were placed into the microtiter wells and incubated for 2 h. After that, the solution was aspirated and washed with 400 µL of washing buffer for four times. Then, 100 µL of caspase-3 was added and incubated for 1 h. The solution in the wells was then replaced with 100 µL of anti-rabbit IgG HRP working solution. After incubation for 30 min in the dark, the wells were washed and incubated for 30 min with 100 µL of stabilized chromogen in the dark. Finally, 100 µL of stop solution was added, and the absorbance at 450 nm was measured using a plate reader (Synergy HT, BioTek, Winooski, VT, USA).

#### 2.9.7. Reactive oxygen species determination

Reactive oxygen species (ROS)/Superoxide detection assay kit (ab139476, Abcam, Cambridge, MA, USA) was used to assess intracellular reactive oxygen species following the manufacturer’s protocol. HepG2 cells were seeded in a black 96-well plate overnight. Then, cells were treated with IC_50_ for 48 h. After that, cells were washed with washing buffer and incubated with an oxidative stress detection solution for 1 h at 37 °C in the dark. ROS fluorescence product was detected by microplate reader (Ex/Em = 495/529 nm) (Synergy HT, BioTek, Winooski, VT, USA).

#### 2.9.8. Mitochondrial membrane potential

Mitochondrial membrane potential **(**MMP) was examined utilizing TMRE Mitochondrial Membrane Potential Assay Kit (Cat. No. ab113852, Abcam, Cambridge, UK). HepG2 cells were seeded in clear-bottom black 96-well plates at a density of 1 × 10^5^ cells per well. The cells were then exposed for 48 h to the IC_50_ concentrations for each treatment. After that, the cells were incubated for 30 min at 37 °C with 200 nM tetramethylrhodamine methyl ester. Finally, the cells were washed with 100 µL of PBS/0.2% BSA and the fluorescence intensity was detected by a microplate reader (Synergy HT, BioTek, Winooski, VT, USA) at Ex/Em = 549/575 nm.

#### 2.9.9. Real-time polymerase chain reaction

##### 2.9.9.1. RNA extraction

RNA was obtained from HepG2 cells utilizing Qiagen’s RNeasy Mini Kit (Qiagen, Manchester, UK) based on the provided instructions. The purity and concentration of RNA were determined using a Nanodrop spectrophotometer (ND-2000C, Thermo Fisher Scientific, Waltham, MA USA). A ratio of A260nm/A230nm of 1.8 and A260nm/A280nm ratio of 1.9 were confirmed in all RNA samples.

##### 2.9.9.2. cDNA Synthesis and qPCR Amplification

RNA samples were reverse-transcribed to complementary DNA (cDNA) utilizing the iScript™ One-Step real-time polymerase chain reaction (RT-PCR) Kit with the SYBR® Green Kit (Bio-Rad, Hercules, CA, USA) based on the provided instructions. Relative expression patterns of *BAX, BCL2, CYCS* and *GAPDH* were assessed using the 7500 Fast Real-Time PCR System (Applied Biosystems, Thermo Fisher Scientific Corporation Waltham, MA, USA) according to the manufacturer’s recommendations. Primer nucleotide sequences are shown in ***Table 3***.

**Table 3.** RT-qPCR primer sequences.

Gene	Accession Number	Primer Direction	Sequence (5’–>3’)
*BAX*	XM_047439168.1	Forward	TCAGGATGCGTCCACCAAGAAG
		Reverse	TGTGTCCACGGCGGCAATCATC
*BCL2*	XM_047437733.1	Forward	ATCGCCCTGTGGATGACTGAGT
		Reverse	GCCAGGAGAAATCAAACAGAGGC
Cytochrome C (*CYCS*)	NM_018947.6	Forward	AAGGGAGGCAAGCACAAGACTG
		Reverse	CTCCATCAGTGTATCCTCTCCC
*GAPDH*	NM_001357943.2	Forward	GAAGGTGAAGGTCGGAGT
		Reverse	GAAGATGGTGATGGGATTTC

### 2.10. Statistical analysis

Data are presented as mean ± standard deviation (SD). Unless otherwise indicated, statistical significance was calculated by one-way analysis of variance (ANOVA) with Tukey’s post hoc test. Differences among samples were regarded as statistically significant at a *p* value <.05. All analyses were conducted using GraphPad Prism software version 8.0.2 for Windows (GraphPad Software Inc., San Diego, CA, USA).

## 3. Results

### 3.1. Characterization of AG

The structure of AG was confirmed by NMR (^1^H and ^13 ^C NMR) and mass spectrometry and its comparison with the spectroscopy data is available in the literature (Du et al., 2003; Akowuah et al., 2006; Syukri et al., 2018).

^1^H NMR (300 MHz, DMSO-d_6_, ppm): *δ* 6.63 (1H, dd, *J* = 4.0, 8.0 Hz), 5.73 (1H, d, *J* = 6.0 Hz), 5.06 (1H, d, *J* = 4.5 Hz), 4.91 (2H, brs), 4.81 (1H, s), 4.62 (1H, s), 4.42–4.37 (1H, m), 4.15–4.13 (1H, m), 4.05–4.01 (1H, m), 3.83 (1H, d, *J* = 10.0 Hz), 3.36–3.23 (2H, m), 2.34–1.18 (11H, m), 1.10 (3H, s), 0.67 (3H, s).

^13^C NMR (100 MHz, DMSO-*d*_6_, ppm): *δ* 170.44, 148.06, 146.79, 129.45, 108.73, 78.92, 74.81, 64.99, 63.12, 55.95, 54.83, 42.75, 37.97, 36.98, 28.36, 24.43, 23.54, 15.22.

ESI MS (+ve): *m*/*z* 351.5 (*M* + 1).

### 3.2. Model fit statistics

Fit statistical analysis result for the particle size is demonstrated in ***Table 4***. Based on the lowest PRESS and the highest *R*^2^, the data of particle size fitted the quadratic model. The adjusted *R*^2^ and the predicted *R*^2^ exhibited appropriate coincidence. Furthermore, the proposed model exhibited an adequate precision value of 21.14.

**Table 4.** Model fit statistics of AG-PTM measured particle size.

Model	Sequential *p* value	Lack of fit *p* value	SD	*R* ^2^	Adjusted *R*^2^	Predicted *R*^2^	PRESS
Linear	.0071	.0042	62.07	0.6279	0.5534	0.2120	81584.80
2FI	.4408	.0035	63.19	0.6529	0.5372	0.7271	1.788 × 10^5^
Quadratic	.0002	.1732	21.37	0.9691	0.9471	0.7739	23412.15

AG, andrographolide; PTM, Phytosomes; 2FI, Two-factor interaction; SD, standard deviation; PRESS, predicted residual error sum of squares.

**Figure 1.**
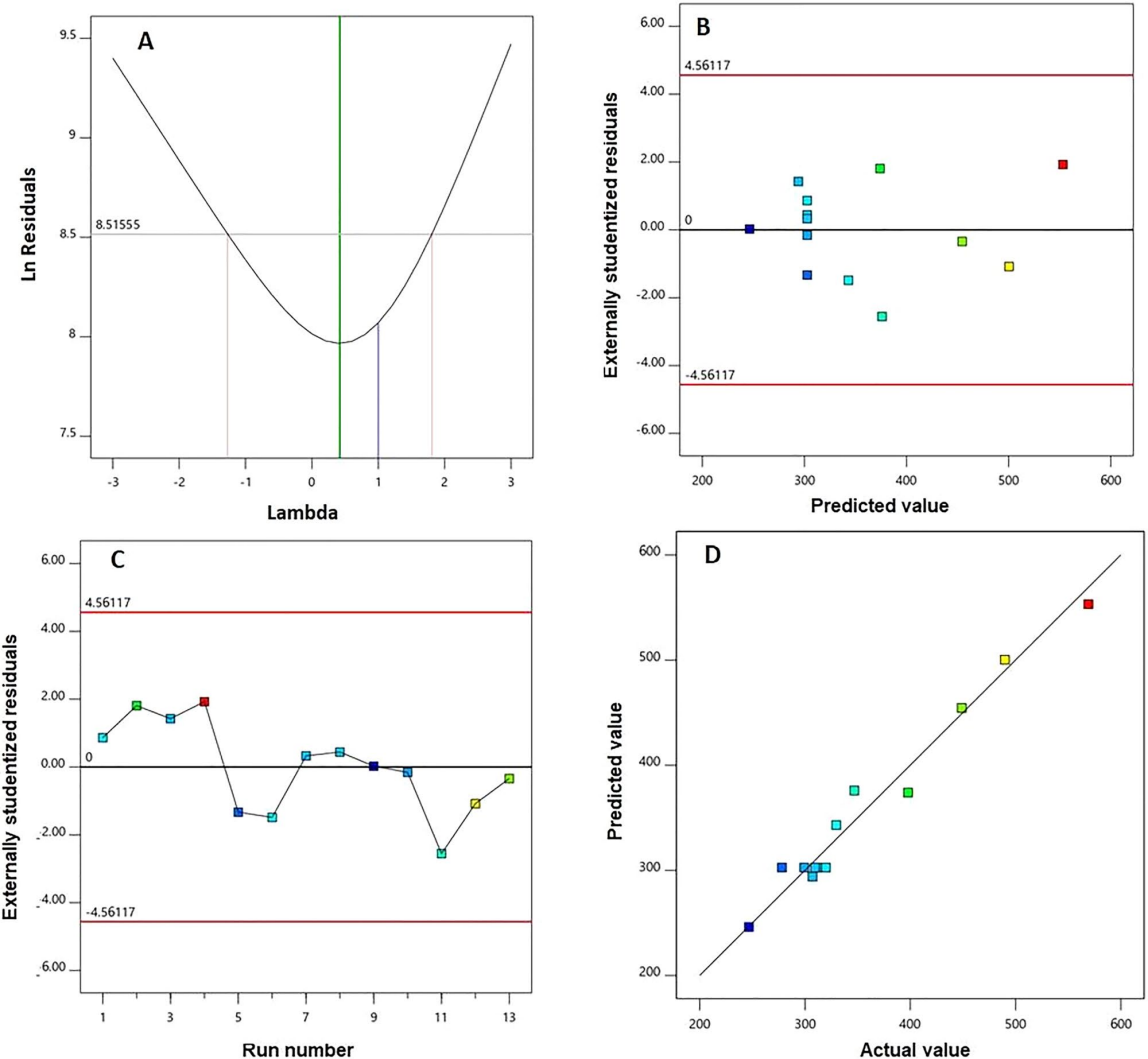
Diagnostic plots for particle size of AG-PTMs. (A) Box-Cox plot for power transforms, (B) externally studentized residuals vs. predicted values plot, (C) externally studentized residuals vs. run number plot, and (D) predicted vs. actual values plot.

The goodness of fit of the quadratic model was also examined through development of diagnostic plots illustrated in ***Figure 1***. Figure 1(A) representing the Box-Cox plot for power transforms shows the best lambda (*λ*) value of 0.42. The 95% confidence limits (shown by the red lines) include the current *λ* value of 1. Externally studentized residuals versus predicted response values, displayed in Figure 1(B), and residual vs. run plots, Figure 1(C), showed randomly distributed points. The predicted versus observed size plot reveals a considerable linear correlation as depicted in Figure 1(D).

### 3.3. Influence of variables on particle size (Y)

The significance of the quadratic model was confirmed by analysis of variance (ANOVA) for size as evidenced by the *F*-value of 43.49 (*p* < .0001), ***Table 5***. A non-significant lack of fit was shown by the lack of fit *F*-value of 2.79 (*p* = .1732); thus, the fitting of the measured size to the proposed model is confirmed.

**Table 5.** Analysis of variance of AG-PTMs measured size according to the quadratic model.

Source	Sum of Squares	DF	Mean Square	*F* value	*p* value
Model	1.003 *E* × 10^5^	5	20067.77	43.94	<.0001
X1: AG:PC molar ratio	5.42	1	5.42	0.0119	.9164
X2: Sonication time (min)	65000.04	1	65000.04	142.31	<.0001
X1X2	2595.90	1	2595.90	5.68	.0486
X12	14440.72	1	14440.72	31.62	.0008
X22	6286.41	1	6286.41	13.76	.0076
Residual	3197.22	7	456.75		
Lack of Fit	2164.04	3	721.35	2.79	.1732
Pure Error	1033.18	4	258.29		
Cor Total	1.035 × 10^5^	12			

AG, andrographolide; PTM, Phytosomes; PC, phosphatidyl choline; DF, degrees of freedom.

**Table 6.** List of IC_50_ values of AG and AG-PTMs against different cancer cell lines.

Treatment	IC_50_ (µM) HCT116	IC_50_ (µM) PC3	IC_50_ (µM) HepG2
AG	9.80 ± 3.90	10.40 ± 3.60	14.09 ± 1.80
AG-PTMs	5.80 ± 3.10	6.80 ± 4.50	4.02 ± 0.14

The equation demonstrating the quadratic model based on the coded factor was produced by the software as follows:

*Y* (particle size) = 302.67 – 0.95 *X*_1_ – 104.08 *X*_2_ + 25.48 *X*_1_*X*_2_ + 72.31 $X_{1}^{2}$ + 47.71 $X_{2}^{2}$

**Figure 2.**
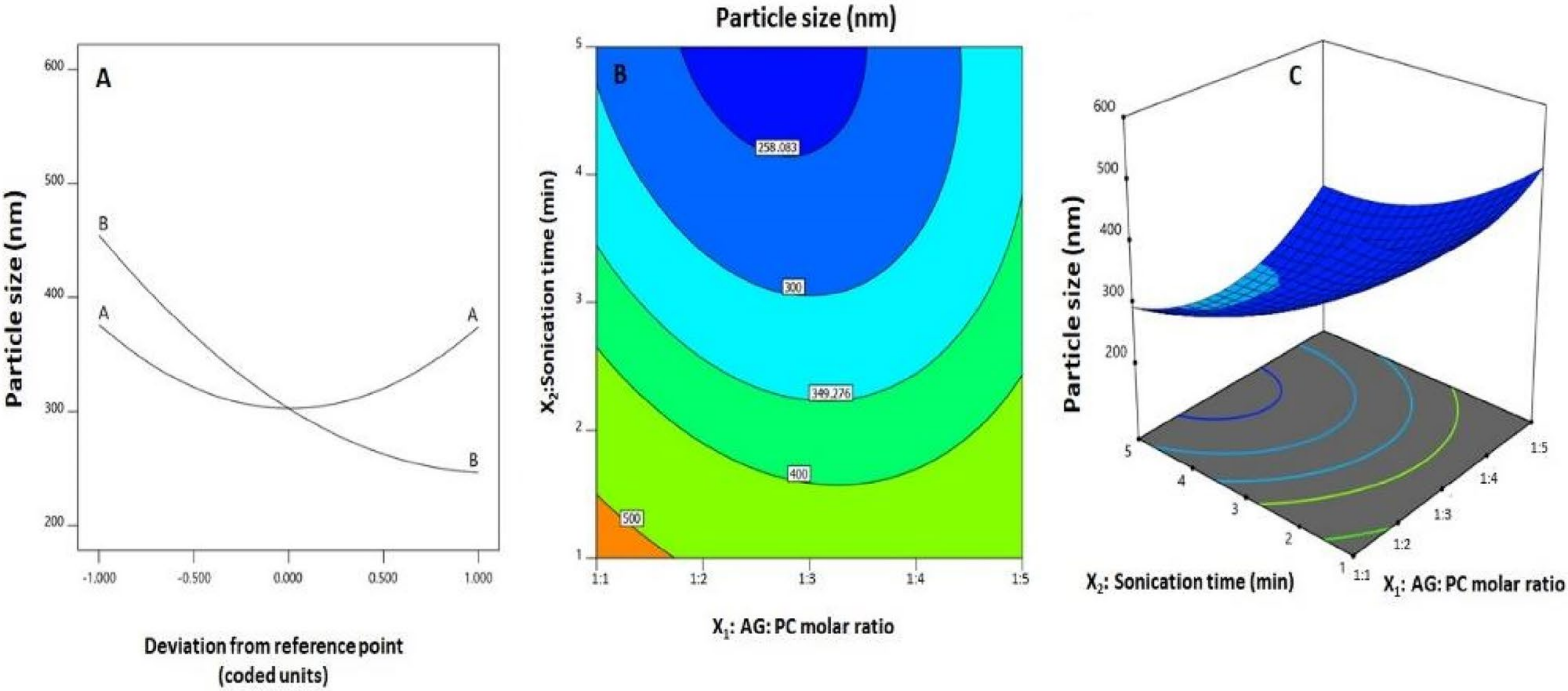
(A) Perturbation, (B) 2D-contour and (C) 3D-surface plots for the effects and interactions between AG: PC molar ratio **(***X*_1_**)** and sonication time (*X*_2_) on the AG-PTM size.

The statistical analysis showed that linear term *X*_2_ corresponding to the sonication time was significant at *p* < .05 as shown in Table 5. Moreover, both quadratic terms ($X_{1}^{2}$ and $X_{2}^{2}$) corresponding to the two investigated variables as well as the interaction term *X*_1_*X*_2_ corresponding to the interaction between them were also significant at the same level. ***Figure 2***(A) demonstrates the perturbation graph indicating the impact of the studied factors on the size, while Figure 2(B,C) shows the 2D-contour and the 3D-response surface plots that demonstrate the interaction between the two variables. The illustrations indicate that the nanosphere size significantly decreases with increasing sonication time. This finding is in harmony with the negative sign of the *X*_2_ coefficient. In addition, the comparatively high value of the aforementioned linear term coefficient in the developed equation indicates that the sonication time is the most influential factor on the size.

### 3.4. Optimization of AG-PTMs

The desirability approach with numerical optimization was utilized to anticipate the optimized variables’ levels that, when combined, can lead to minimized size. The optimized levels of variables were 1:2.7 for AG:PC molar ratio and 4.9 min for sonication time, respectively. The measured particle size of 243.70 ± 7.30 nm with polydispersity index (PDI) of 0.31 coincides well with the predicted one (244.91 nm) showing a relative percentage error of 0.494%. In this regard, the reliability of the optimization process is highlighted by the relatively small percentage errors. In addition, our data indicated that entrapment efficiency AG in AG-PTMs was found to be 72.20 ± 4.53%.

### 3.5. FTIR characterization of the optimized AG-PTMs

**Figure 3.**
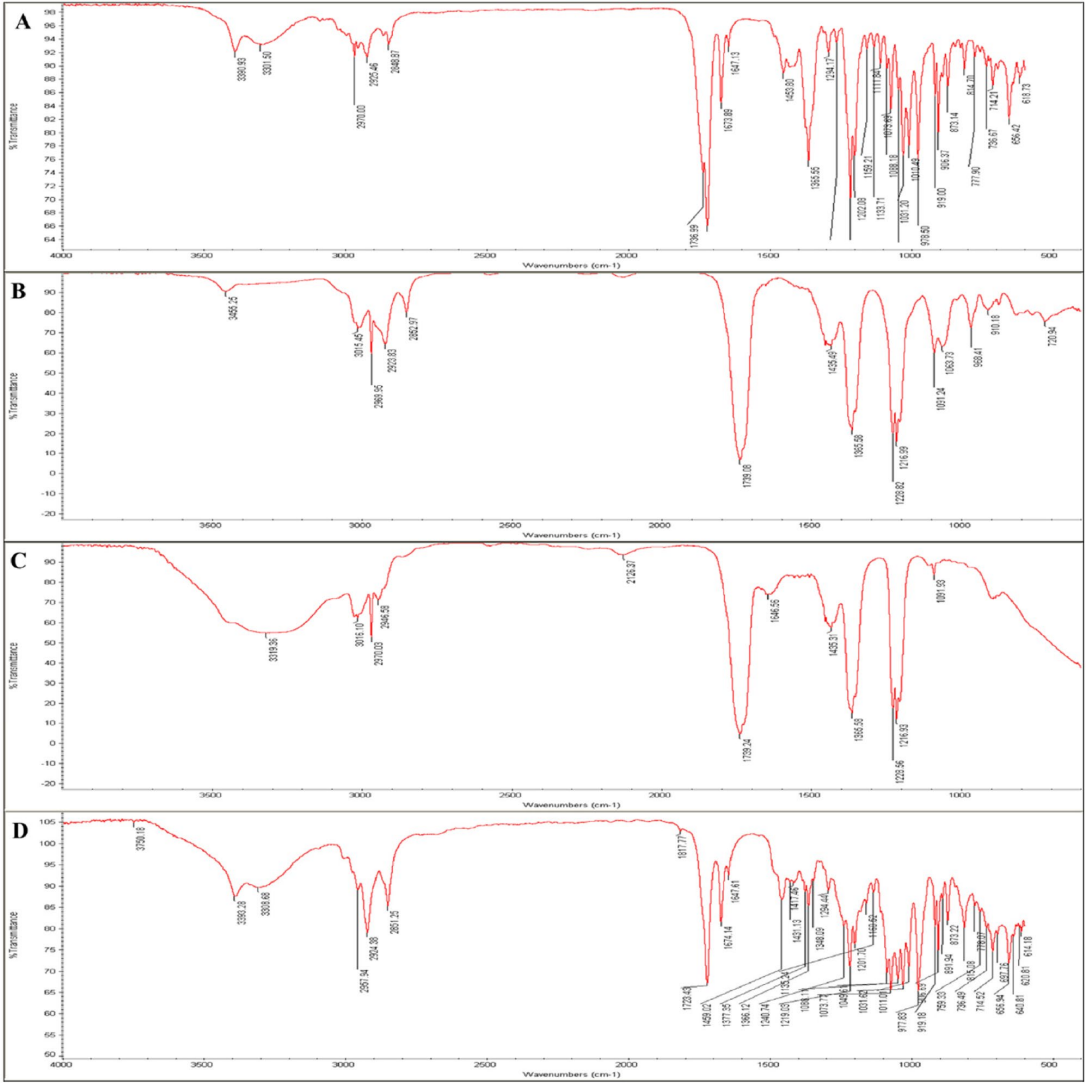
Fourier-transform infrared (FTIR) of AG (A), PC (B), AG-PTMs (C) and a simple mixture of AG and PC (D).

FTIR spectroscopy was used to investigate the interaction between components of the optimized AG-PTMs. As shown in ***Figure 3***(A), the most characteristic IR bands for AG are the broad bands at 3301–3390 cm^−1^ for alcoholic hydroxyl groups (-OH groups). In addition, the characteristic absorption band at 2970 cm^−1^ is related to the aliphatic -CH_2_- stretching vibration. The sharp peak at 1736 cm^−1^ corresponds to the carbonyl group (-C = O group) of the cyclic ester moiety (lactone). The peaks at 1673 cm^−1^ for the C = C double bond and 1294 cm^−1^ for the C-O of cyclic ester were also detected in the spectrum. The FTIR spectra of PC showed the aliphatic -CH_2_- stretching band at 2969–3015 cm^−1^. The two carbonylic esters groups (2 C = O) appear as two bands at 1739 cm^−1^. In addition, the peak at 1216 cm^−1^ represents C-O vibration. A P = O stretching peak at 1228 cm^−1^, P-O-C stretching band at 1091 cm^−1^ and -N+(CH_3_)_3_ stretching band at 968 cm^−1^ were also assigned in the spectrum (Figure 3(B)). The FTIR spectrum of AG-PTMs revealed the interactions of the two components, AG, and PC, as shown in Figure 3(C). The broadness and intensity of the OH group peaks at 3319 cm^−1^ augmented, which may be due to the involvement of the -OH groups by more intermolecular H-bonding between AG and PC, this may be attributed to the interactions between the three hydroxyl groups of AG (H-bond donors) get involved in more H-bonding of electronegative O-atoms of esters of PC (H-bond acceptor). Moreover, the aliphatic -CH_2_- groups of both AG and PC are still represented at 2970–3016 cm^−1^, and they may be packed together by non-polar forces e.g., Van der Waal forces. The broadness and strong peak at 1739 cm^−1^ may refer to the complete overlap between two regions of 3 carbonylic esters (3 C = O) of AG and PC. Thus, they also, participate in the stabilization of intermolecular H-bonding as an H-bond acceptor. For the FTIR spectrum of a simple mixture of AG and PC with the same molar ratio as in our formula, it was noticed that the distinctive peaks for pure AG and PC were present without any change or increase in peaks broadness and intensity e.g., at OH group region at 3319 cm^−1^ and -CH_2_- groups at 2970–3016 cm^−1^. On the other hand, the fingerprint bands of AG and PC were present. In conclusion, the spectrum excludes any chemical interaction between AG and PC ingredients without formulation.

### 3.6. Ag release from AG-PTMs

**Figure 4.**
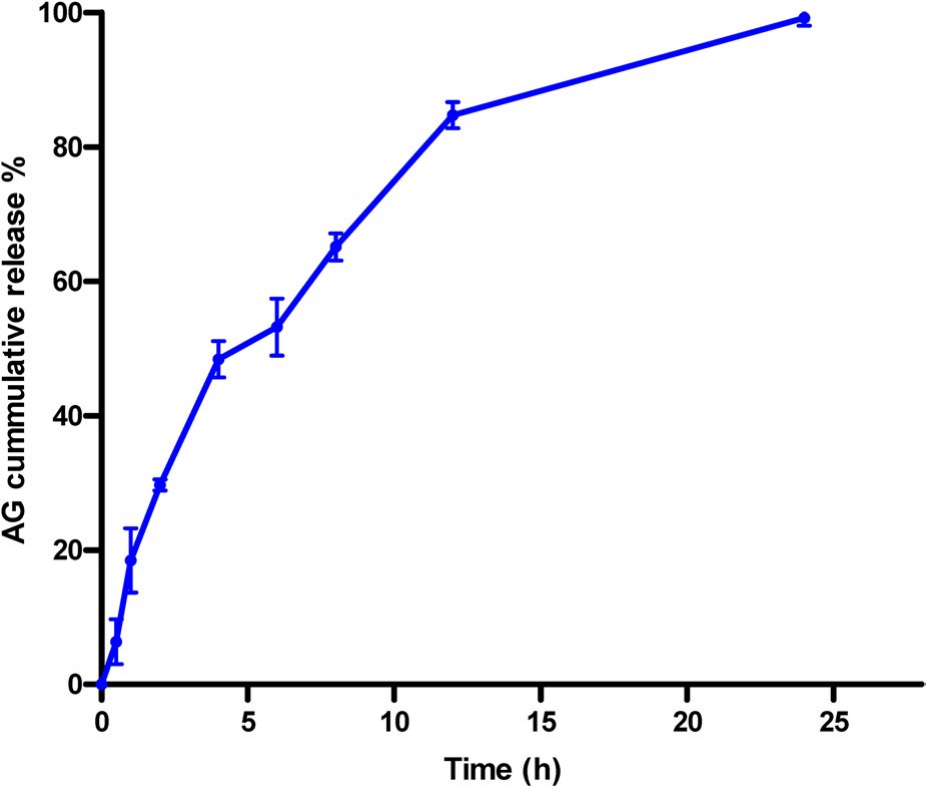
In vitro release pattern of optimized AG-PTMs after 24 h.

Analysis of in vitro AG release from the optimized AG-PTMs indicated sustained release pattern (***Figure 4***). It shows that 84.72 ± 1.95 and 99.18 ± 1.14 of AG were released from AG-PTMs at 12 and 24 h, respectively

### 3.7. Pharmacological effects of optimized AG-PTMs on HepG2 cells

#### 3.7.1. Cell viability assay

**Figure 5.**
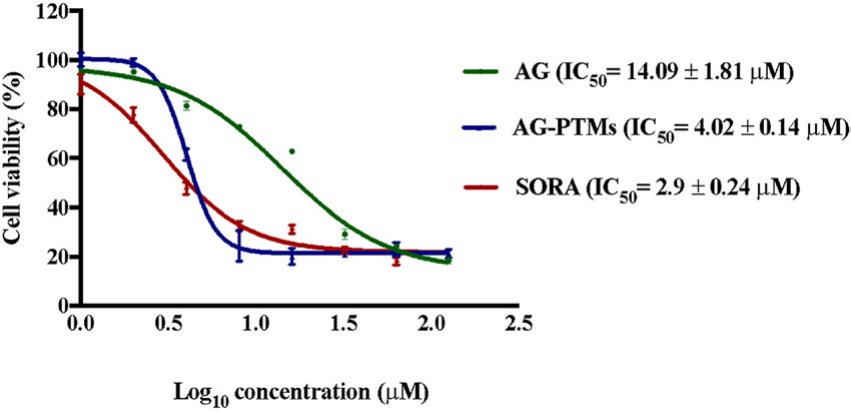
Antiproliferative effects of AG, AG-PTMs and SORA in HepG2 cells. Data are represented as mean ± SD of six independent experiments.

MTT assay was utilized to investigate the antiproliferative effect of AG, and AG-PTMs against several cancer cell lines. Our data indicated that AG-PTMs produced the highest antiproliferative activities against HepG2 cells (Table 1). Therefore, this cell line has been selected to further analyze the antiproliferative of the new formulation. The effect of blank-PTMs, AG, AG-PTMs and SORA on HepG2 cells after an incubation period of 48 h is shown in (***Figure 5***). AG showed a concentration related antiproliferative effect with IC_50_ value of 14.09 ± 1.81 µM (*p* < .05). Blank-PTMs did not exert any antiproliferative activity against HepG2 cells at all concentrations. Treating the cells with AG-PTMs exhibited a 3.5-fold reduction in viability (IC_50_ 4.02 ± 0.14 µM) when compared to the AG (*p* < .05). A significant cytotoxic activity was detected in SORA-treated cells showing an IC_50_ value of 2.90 ± 0.24 µM (Table 6). These data suggest that the AG-PTMs significantly reduced the viability of HepG2 cells and this optimized formulation enhanced the antiproliferative activity of AG.

#### 3.7.2. Cellular uptake

**Figure 6.**
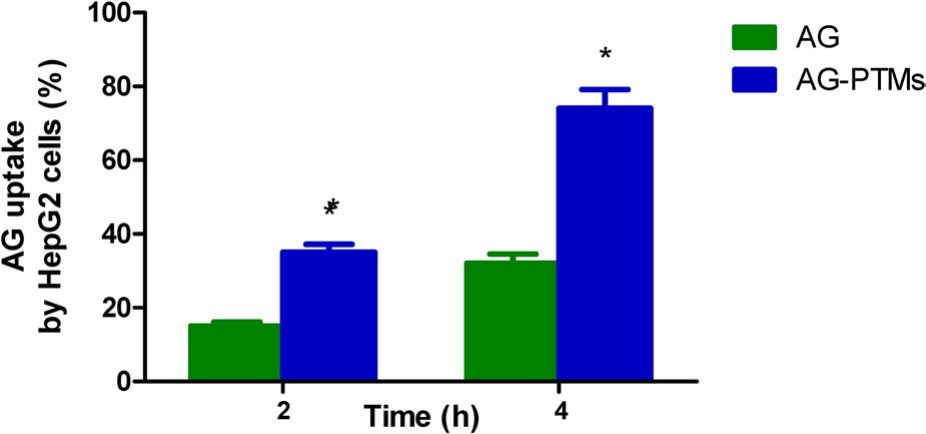
Cellular uptake of AG by HepG2 cells. *Significantly different from corresponding AG at *p* < .05 as determined by independent Student *t* test.

***Figure 6*** shows that cellular uptake of Ag from AG-Raw attained values of 15.20 ± 1.10% and 32.10 ± 2.50% at 2 and 4 h after incubation, respectively. Significantly elevated cellular uptakes were observed in incubations containing AG-PTMs, which reached 35.30 ± 2.20% and 74.00 ± 5.10% at 2 and 4 h after incubation, respectively.

#### 3.7.3. Cell cycle analysis

**Figure 7.**
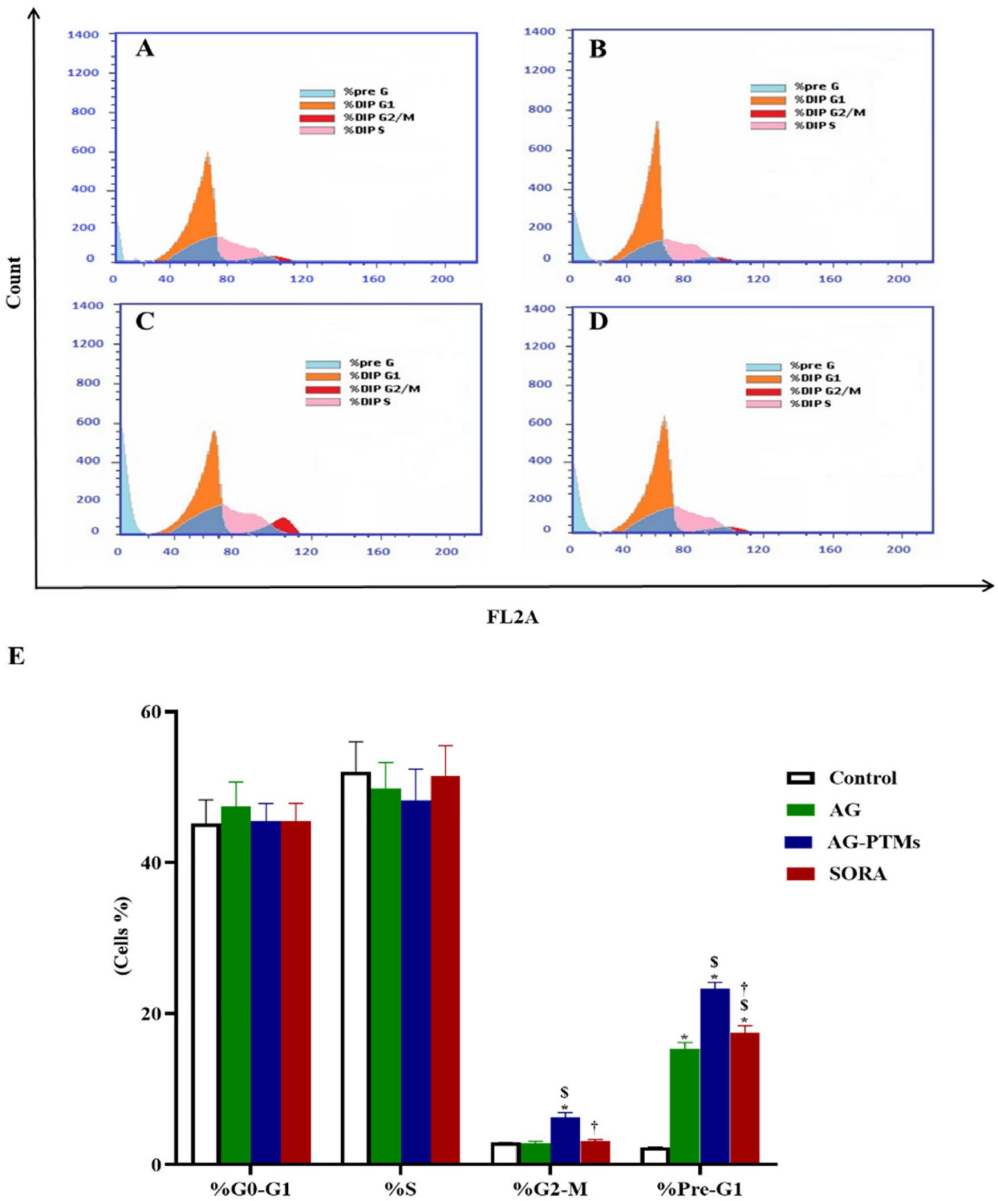
Cell cycle analysis of HepG2 cells, (A) Control cells or pretreated with (B) AG, (C) AG-PTMs and (D) SORA. (E) Representative bar chart demonstrating the proportions in the cell cycle phases. Data are represented as mean ± SD of six independent experiments. *A statistically significant difference from control at *p* < .05, $statistically significant difference from AG at *p* < .05, †statistically significant difference from AG-PTMs at *p* < .05.

The antiproliferative effect of AG-PTMs against HepG2 was further evidenced by cell cycle analysis. As presented in ***Figure 7***(A,E), control cells exhibited a proliferative profile with 45.13% at G0-G1 phase, 52% at S phase, 2.87% at G2-M phase and 2.25% at pre-G1 phase. Yet, treatment of cells with AG caused a significant rise in apoptotic cells in the pre-G1 phase relative to the control (*p* < .05, Figure 7(B,E). SORA-treated cells also revealed an accumulation of cells in the pre-G1 phase in a comparable manner to AG (Figure 7(D,E)). Interestingly, treating the cells with AG-PTMs significantly increased the G2-M cell population when compared to control, AG and SORA (*p* < .05) (Figure 7(C,E)). In addition, AG-PTMs-treated cells showed a significant increase in the pre-G1 apoptotic cell fraction when compared to control, AG and SORA (*p* < .05). These results suggested that AG-PTMs arrested the cell cycle at the G2-M and pre-G1-phases at a very low concentration.

#### 3.7.4. Assessment of Annexin V staining

**Figure 8.**
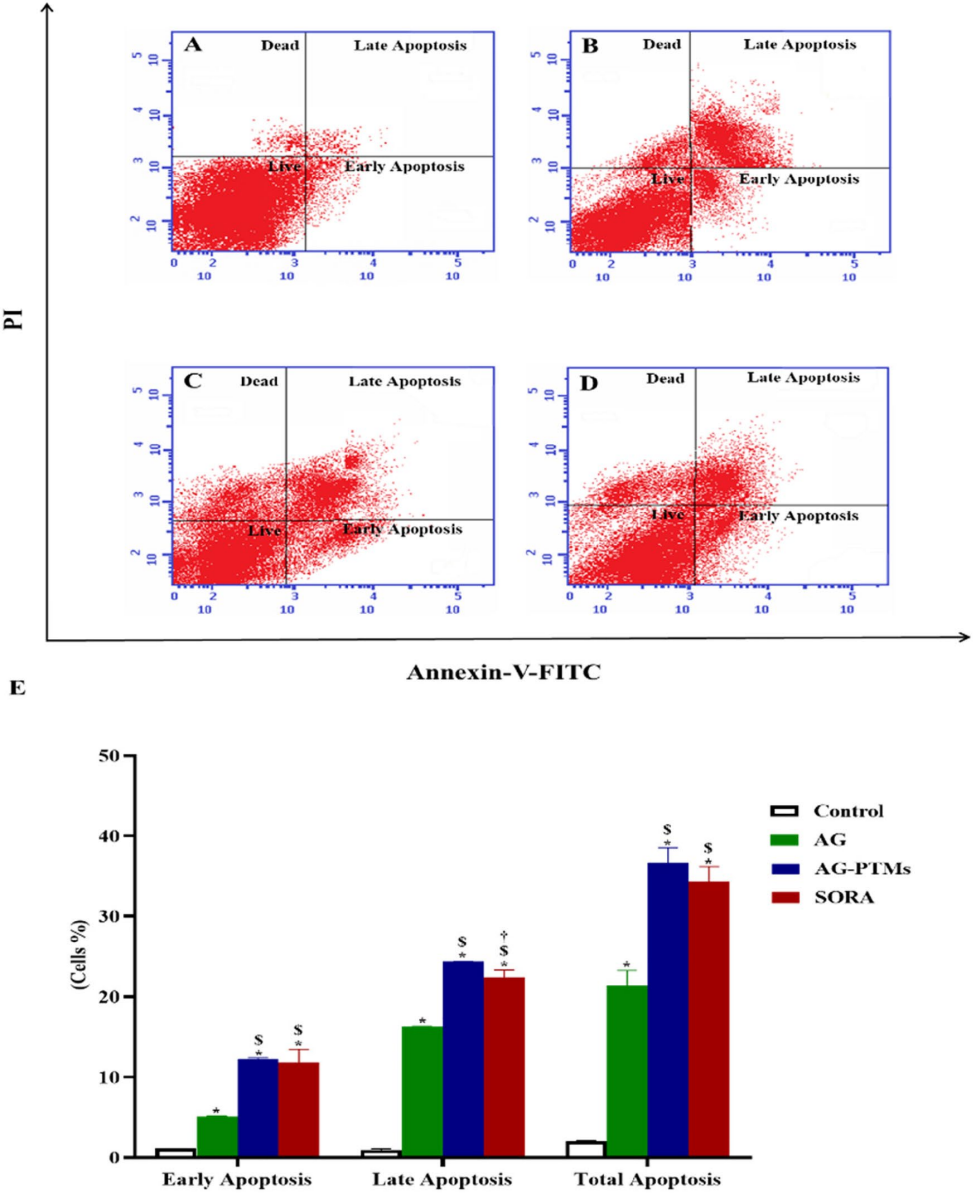
Detection of apoptosis by flow cytometry in HepG2 cells pretreated with AG, AG-PTMs and SORA. Representative flow cytometric dot plots of control (A), AG (B), AG-PTMs (C) and SORA (D). Graphic presentation of early, late and total apoptosis (E). Data are represented as mean of six independent experiments ± SD. *Statistically significant differences from control at *p* < .05, $statistically significant differences from AG at *p* < .05, †statistically significant differences from AG-PTMs at *p* < .05.

Since AG-PTMs induced pre-G1 phase arrest in HepG2 cells, the Annexin-V-FITC/PI assay was utilized to confirm this apoptotic effect. As presented in ***Figure 8***, all treatments significantly induced early, late and total apoptosis as compared to the control cells (*p* < .05). Interestingly, AG-PTMs induced a significant increase in HepG2 apoptotic potential when compared to AG (*p* < .05), indicating that AG-PTMs improved AG’s ability to induce apoptosis. It is well noted that SORA exhibited no significant changes in the fraction of total apoptotic cells as compared to AG-PTMs (Figure 8(A–E)). This apoptotic activity of AG-PTMs against HepG2 cells was further confirmed by studying its effect on several apoptotic markers.

#### 3.7.5. Effect of AG-PTMs on mitochondrial membrane potential, generation of reactive oxygen species and mRNA expression of *CYSC*

**Figure 9.**
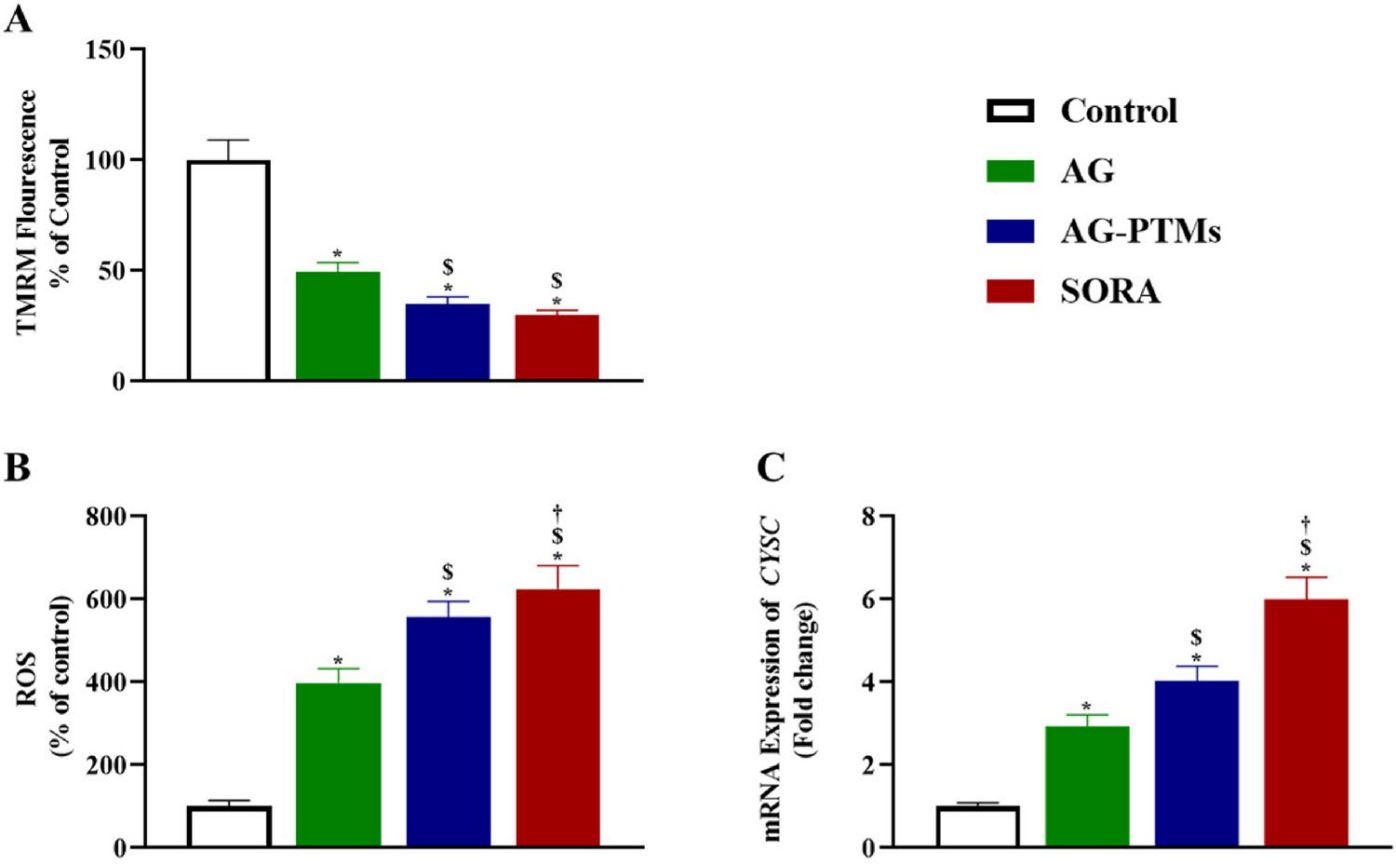
Graphic presentation of changes in MMP (A), ROS production (B) and mRNA expression of *CYSC* (C) in HepG2 cells pretreated with AG, AG-PTMs, and SORA. Data are represented as mean of six independent experiments ± SD. *Statistically significant differences from control at *p* < .05, $statistically significant differences from AG at *p* < .05, †statistically significant differences from AG-PTMs at *p* < .05.

As shown in ***Figure 9***(A), treating cells with AG-PTMs showed a significant loss of mitochondrial membrane potential (MMP) (35.09 ± 3.10) compared to control (100.00 ± 9.00), and AG (49.54 ± 4.20) (*p* < .05). To determine whether the loss of MMP is associated with increased ROS production, which is known as an early sign of apoptosis, we measured ROS production in HepG2 cells upon exposure to all treatments. As presented in Figure 9(B), AG-PTMs significantly increased the level of ROS in HepG2 cells (557.00 ± 38.25) when compared to control (100.00 ± 14.58), and AG (398.50 ± 34.05) (*p* < .05). In addition, subjecting HepG2 cells to AG-PTMs significantly increased the expression of *CYSC* (4.02 ± 0.36) when compared to control (1.00 ± 0.09) and AG (2.93 ± 0.28) (*p* < .05) (Figure 9(C)).

#### 3.7.6. Effect of AG-PTMs on *BAX* and *BCL2* mRNA expression and active caspase-3

**Figure 10.**
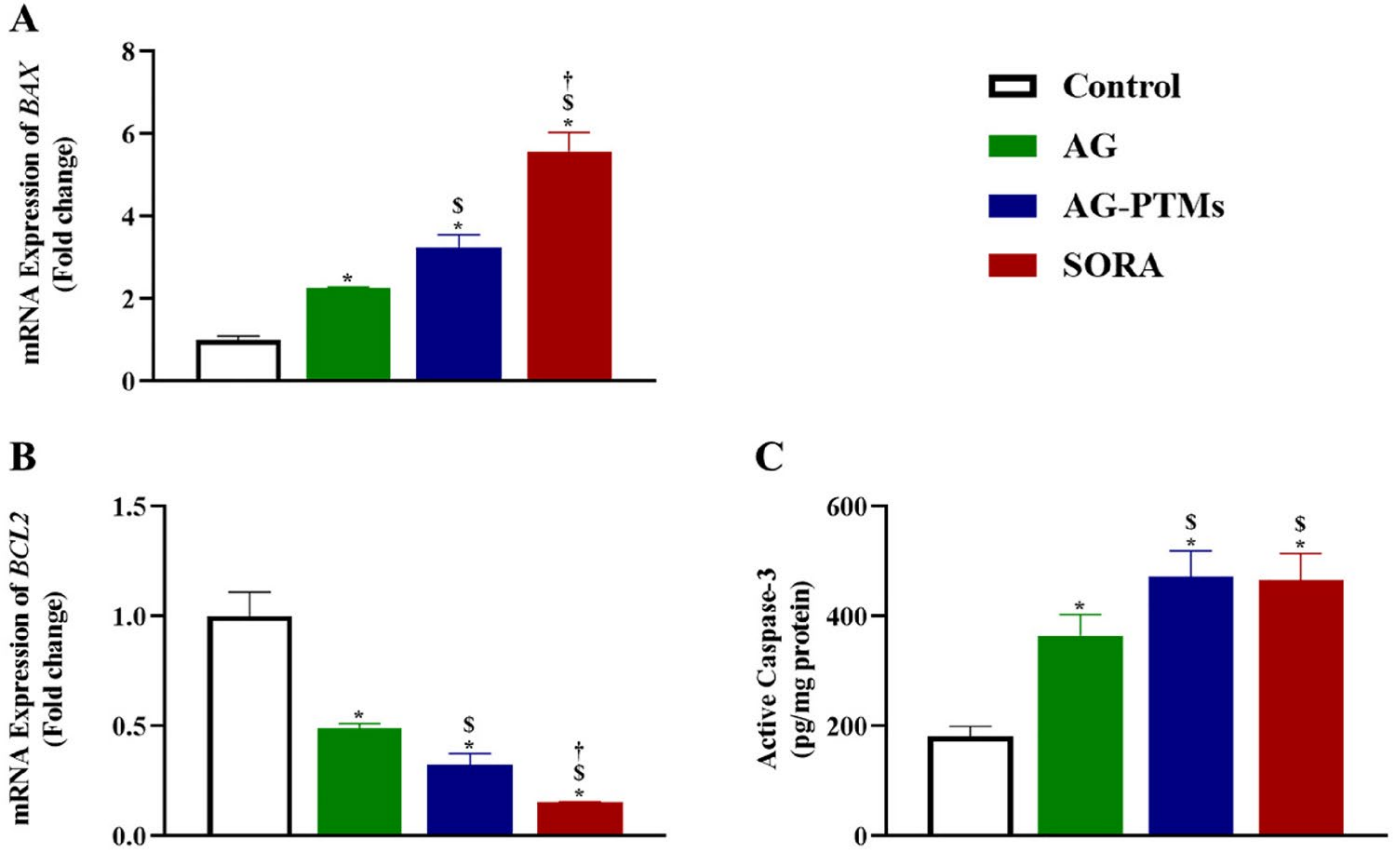
Graphic presentation of *BAX* (A) and *BCL2* (B) mRNA expressions, and active caspase-3 concentrations (C) in HepG2 cells pretreated with AG, AG-PTMs, and SORA. Data are represented as mean of six independent experiments ± SD. *Statistically significant differences from control at *p* < .05, $statistically significant differences from AG at *p* < .05, †statistically significant differences from AG-PTMs at *p* < .05.

The effect of AG-PTMs on the expression of apoptosis-related genes *BAX and BCL2* is presented in ***Figure 10***. The mRNA level of the pro-apoptotic *BAX* was significantly upregulated after exposure to AG-PTMs (3.24 ± 0.33) when compared to control (1.00 ± 0.10) and AG (2.26 ± 0.03) (*p* < .05). Correspondingly, the anti-apoptotic gene *BCL2* was downregulated with AG-PTMs to 0.32 ± 0.05, as compared to control (1.00 ± 0.11) and AG (0.49 ± 0.02) (*p* < .05) (Figure 10(B)). Furthermore, the expression of caspase-3 protein increased upon AG-PTMs treatment (471.60 ± 48.31), when compared to the control (181.80 ± 17.90) and AG (364.50 ± 38.57) (*p* < .05). It is worth noting that the active caspase-3 concentration in cells treated with AG-PTMs was comparable to that observed in the SORA-treated cells (466.30 ± 48.04) (Figure 10(C)). These results indicate that apoptosis induction in HepG2 cells was dependent on caspase-3 activity, and mainly mediated via the intrinsic pathway with increased ROS production.

## 4. Discussion

Liver cancer is a leading cause of cancer-related mortalities. AG is known to inhibit the proliferation of cancerous liver cells (J. Li et al., 2007; Ooi et al., 2011; W. Chen et al., 2012; Chowdhury et al., 2019). However, the strong hydrophobic property of AG could limit its clinical use. In this regard, nano-formulated AG has been proposed to overcome this issue, hence a face-centered central composite design was employed in this study to formulate AG-loaded PTMs. The optimum combination of the independent variables; AG:PL ratio and ST, was determined to achieve the minimum size. The difference between the adjusted *R*^2^ and the predicted value was less than the permissible limit of 0.2 which highlights the model’s validity. In addition, the adequate precision of the proposed model was greater than the desired value of 4 demonstrating a suitable signal-to-noise ratio. Based on the parameters computed previously, the quadratic model can be effectively employed to investigate the experimental design space (Al-Mahallawi et al., 2015; Aldawsari & Badr-Eldin, 2020). The inclusion of the current *λ* within the 95% confidence limits in the Box-Cox plot for power transformation resulted in no suggested, specific transformation for the observed size (Badr-Eldin et al., 2021; Singh et al., 2011). The randomly scattered points in the externally studentized residuals versus predicted response plot within the limits indicate no existing constant error. Furthermore, the random distribution of points in the residual vs. run plot and the absence of trend indicate that no lurking variable would affect the determined size. In addition, the highly linear correlation of the predicted versus observed size reflects the harmony between the observed and predicted values indicating the appropriateness of the model (Fahmy, Badr-Eldin et al., 2020). The experimental design results showed that sonication time in the current study exhibited a similar negative effect on the particle size, indicating that the particle size significantly decreases with increasing sonication time (Alhakamy et al., 2022). The observed reduction in size could be attributed to the main principle of the sonication process. The ultrasound mechanical waves of sonication lead to cavitation bubbles formation in the phytosomal dispersions. Bubbles with size close to the resonant size of the employed frequency start to oscillate nonlinearly causing bubble breakdown; this action induces extremely high temperature and pressure, as well as generates shock waves. Thus, the ultrasonic generated high energy leads to size reduction. Based on this interpretation, the developed energy increases at a higher sonication time resulting in particle size reduction (Essa, 2010; Harbi et al., 2016; Fahmy, Aldawsari et al., 2020). Most of the prepared AG-PTMs exhibited promising sizes that ranged from 246.7 to 569.2 nm. Particulate delivery systems with a size smaller than 400 nm have recently gained popularity in the field of cancer therapy owing to their ability to be preferentially distributed within solid tumors (Sharma et al., 2014; Yingchoncharoen et al., 2016). Despite this finding, it is reported that the accumulation of the nanoparticulate systems and consequently their clinical efficacy might be prevailed over by inefficacious cancerous tissue penetration, which could occur due to the pathological manifestations created by the malignancy development (Zhang et al., 2019). However, tumor penetration could be enhanced by decreasing the size and simultaneously increasing the available surface area for cell penetration (Badr-Eldin et al., 2021). Thus, the study goal was to optimize the developed PTMs to minimize size.

The current study indicated that the prepared optimized AG-PTMs had a relatively good entrapment efficiency (>70%) and appreciable slow release pattern. Both AG and AG-PTMs had a significant cytotoxic effect against HepG2 cells. In consistent with previous reports, AG has shown to inhibit the viability of HepG2 cells resulting in an IC_50_ range of (25–40.2 μM) (J. Li et al., 2007; Tu et al., 2014). Here in, AG-PTMs significantly suppressed the proliferation of HepG2 cells when compared to raw AG. This indicates that loading AG into PTMs enhanced its cytotoxicity at a considerably low concentration. Furthermore, our data indicated that AG-PTMs showed significantly higher uptake by HepG2 cells. Thus, the superior effect of AG-PTMs could be attributed to its favorable cellular penetration due to its nanosized particles with greater surface area, thus AG easily enter the cancerous cells to exert it pharmacological effect. This is supported by the reports indicating that cellular uptake of polymeric nanoparticles is enhanced by an endocytosis (Conner & Schmid, 2003). The use of PTMs to enhance the cytotoxicity of phytochemical compounds such as sinigrin, quercetin and curcumin has been documented earlier (Alhakamy et al., 2021; Al-Rabia et al., 2022; Mazumder et al., 2016). Therefore, our findings primarily confirm the advantage of loading AG into PTMs to enhance the antiproliferative effect of AG.

Cell cycle analysis further confirmed AG-PTMs cytotoxicity against HepG2 cells. Our phytosomal formulation accumulated HepG2 cells at G2-M phase. Similarly, it was shown that AG can induce G2-M arrest in human melanoma C8161, A375 and human glioblastoma U251 and U87 cell lines (G. Liu & Chu, 2018; Y. Li et al., 2012). AG-PTMs significantly arrested mitosis when compared to pure AG, which mostly resulted in cellular apoptosis. This was confirmed by the significant increase in pre-G1 apoptotic cell population. Cell accumulation at the G2-M phase also suggests DNA damage which is difficult to repair (Lezaja & Altmeyer, 2018). Myra et al. have previously referred to the antiproliferative activity of AG in HepG2 cells due to blockage of the cells specifically at metaphase (Cheung et al., 2012). Another work documented AG-induced mitotic blockage was confirmed by Cdc2 downregulation, a key enzyme of G2-M transition (J. Li et al., 2007). The current findings are consistent with these reports; however, our formulation successfully enhanced the antiproliferative effect of AG against HepG2 cell through the significant induction of G2-M arrest.

Induction of apoptosis is often suppressed by several mechanisms in cancer (Pfeffer & Singh, 2018; Wong, 2011). Mitochondrial damage with intracellular ROS generation plays a crucial role in the intrinsic mitochondria-mediated apoptosis pathway (Lemasters, 1999; Crompton, 1999). Loss of MMP alters the expression of BAX and Bcl-2, resulting in the activation of caspases due to the release of cytochrome c from the mitochondria into the cytoplasm (Yang et al., 1997; Kim & Park, 2003; Ly et al., 2003). Here in, our data showed that AG-PTMs enhanced the apoptotic activity of AG as evidenced by the significant increase in early, late and total apoptotic death. Li et al previously reported the apoptotic profile of AG in HepG2 cells. AG has shown to reduce MMP, increases hydrogen peroxide generation and induces the expression of Bax and the activity of caspase-3 in HepG2 cells (J. Li et al., 2007). Furthermore, Banerjee et al. demonstrated that AG increased ROS production, reduced MMP, increased Bax/Bcl-2 ratio and caspase-3 activity in MDA-MB-231 breast cancer cells (Banerjee et al., 2016). In addition, ROS generation is involved in AG-induced apoptosis in various cancer cell lines (S. Wang et al., 2020; Lim et al., 2017). These reports further support our findings that AG-PTMs exerts its activity via the generation of ROS. However, it is important to highlight that AG-PTMs potentially enhanced the induction of apoptosis of AG. Our results demonstrate that AG-PTMs have significantly induced the expression of both *BAX* and *CYCS*, reduced the expression of *BCL2*. Thus, it can be suggested that the observed activity is attributable to enhanced AG cell penetration due to loading it into PTMs. The current work also revealed that AG-PTMs elevated the content of active caspase-3 in HepG2 cells when compared to pure AG. This further supports the observed apoptotic effect of AG-PTMs all over the study. In addition, increasing in caspase-3 concentration is in line with reported apoptotic effect of AG on HepG2 cells and several cancer cells (Lim et al., 2017; Harjotaruno et al., 2007). Considering the neglected effect of PTMs vehicle in each experiment of the study, it can be concluded that the cytotoxic and apoptotic properties of AG-PTMs are attributed solely to AG, which was potentially enhanced due to use of PTMs.

## 5. Conclusions

In this current study, AG-loaded phytosomes were formulated with improved characteristics to enhance the antiproliferative effect of AG against HepG2 cells. This was evidenced by cell cycle arrest at the G2-M phase and the induction of intrinsic mitochondrial-mediated apoptosis.
